# Auriculotherapy for anxiety, quality of life and fear of COVID-19 in pregnant women: a randomized clinical trial

**DOI:** 10.1590/0034-7167-2024-0062

**Published:** 2025-04-28

**Authors:** Hérica Pinheiro Corrêa, Kauê Batista Andrade, Tânia Couto Machado Chianca, Antônio Prates Caldeira, Carolina Amaral Oliveira Rodrigues, Maria Fernanda Santos Figueiredo Brito, Caroline de Castro Moura, Diego Dias de Araújo

**Affiliations:** I Universidade Estadual de Montes Claros. Montes Claros, Minas Gerais, Brazil; II Universidade Federal de Minas Gerais. Belo Horizonte, Minas Gerais, Brazil; III Universidade Federal de Viçosa. Viçosa, Minas Gerais, Brazil

**Keywords:** Auriculotherapy, Anxiety, Quality of Life, COVID-19, Clinical Trial, Auriculoterapia, Ansiedad, Calidad de Vida, COVID-19, Ensayo Clínico

## Abstract

**Objectives:**

to assess the effect of auriculotherapy on anxiety, quality of life and fear of COVID-19 in pregnant women.

**Methods:**

multicenter, single-blind, randomized clinical trial. Fifty-three pregnant women participated in intervention group and 51 in placebo group. Outcomes were measured by State Anxiety Inventory, World Health Organization Quality of Life-Bref, Fear of COVID-19 Scale and intervention assessment. Data were analyzed using Generalized Estimating Equations.

**Results:**

in both groups, there was reduced anxiety. In terms of quality of life, intervention group showed significant improvement in physical and environmental domains. In psychological domain, there was improvement in both groups, and in social domain, there was no significant change. Fear of COVID-19 decreased significantly in both groups.

**Conclusions:**

auriculotherapy had an effect equal to that of the placebo group in reducing anxiety and fear of COVID-19, and superior effect in the quality of life physical and environmental domains. REBEC: RBR-7fsjmyc.

## RESUMEN

**Objetivos:**

evaluar el efecto de la auriculoterapia sobre la ansiedad, la calidad de vida y el miedo al COVID-19 en mujeres embarazadas.

**Métodos:**

ensayo clínico multicéntrico, aleatorizado, simple ciego. 53 mujeres embarazadas participaron en el grupo de intervención y 51 en el grupo de placebo. Los resultados se midieron mediante el Inventario de Ansiedad Estatal, la *World Health Organization Quality of Life-Bref*, la Escala de Miedo Perinatal COVID-19 y la evaluación de la intervención. Los datos se analizaron utilizando *Generalized Estimating Equations*.

**Resultados:**

en ambos grupos hubo reducción de la ansiedad. En calidad de vida, el grupo de intervención mostró una mejora significativa en los dominios físico y ambiental. En el ámbito psicológico, hubo mejoras en ambos grupos, y en el ámbito social, no hubo cambios significativos. El miedo al COVID-19 disminuyó significativamente en ambos grupos.

**Conclusiones:**

la auriculoterapia tuvo un efecto igual al del grupo placebo en la reducción de la ansiedad y el miedo al COVID-19, y un efecto superior en los dominios físico y ambiental de la calidad de vida. REBEC: RBR-7fsjmyc.

## INTRODUCTION

Pregnancy predisposes and makes women more exposed to anxiety due to physiological and psychosocial changes, current and future concerns regarding pregnancy^(1)^. Pregnancy-related anxiety differs from general anxiety disorders in that it is specific to the outcomes of pregnancy, childbirth, postpartum, infant and maternal health^(2)^.

The prevalence of anxiety during pregnancy, described in the world literature, is 18.2% in the first trimester, increasing to 19.1% in the second trimester and 24.6% in the third trimester^(3)^. In Brazil, in a multicenter cross-sectional study, it was observed that, during the COVID-19 pandemic, 16.1% of pregnant women presented moderate maternal anxiety and 11.5%, severe maternal anxiety^(4)^.

Anxiety is a multifactorial condition, and its interaction with the immune, neuroendocrine and placental systems has a significant impact on maternal and fetal health and well-being, with shortand long-term consequences^(5-8)^. Negative impacts include increased risk of negative obstetric outcomes^(9)^, such as intrauterine growth restriction^(10)^, low birth weight, miscarriage, and preterm birth^(11)^.

In the pandemic context, fear of the unknown, associated with expecting a baby, access to information and greater morbidity and mortality from the virus in pregnant women, generated mental illness^(12)^ and a negative impact on pregnant women’s quality of life^(13,14)^. Thus, chronic or inappropriate fear, which is closely related to common mental disorders such as anxiety, has been the target of monitoring and interventions^(13,15)^, in addition to investigations, such as the present study.

The management of emotional and psychological changes, among which anxiety stands out, is carried out in Primary Health Care (PHC)^(16)^. However, the treatment of this condition receives little attention from professionals and pregnant women, due to the perception that anxiety symptoms, even when pathological, are natural to pregnancy and due to the lack of approach to psychosocial aspects^(17)^.

In this context, nurses stand out, given that, after identifying emotional and psychological changes, such as anxiety during pregnancy, they plan and implement adaptive coping strategies^(17)^. Among the interventions, Integrative and Complementary Health Practices (ICHP), especially auriculotherapy, have been widely used by nurses to manage emotional situations^(18,19)^.

In the global scenario, the use of ICHP by the general population varies between 39.3%^(20)^ and 51.8%^(21)^, and it is estimated that one in three pregnant women use them^(22)^. In many situations, non-pharmacological interventions are the first treatment option for anxiety^(10)^. Since ICHP have been considered strategies for promoting health and treating common complaints during pregnancy^(22,23)^, it is important that health professionals are aware of the interventions, considering the possibility of their influence on pregnancy and possible interactions with medications and fetal complications^(22)^.

However, despite the efficacy and safety of auriculotherapy for anxiety in the general population^(18)^, there is a lack of studies on this intervention in pregnant women in the prenatal period^(24)^ as well as those designed to assess fear of COVID-19 and quality of life of pregnant women in the context of the COVID-19 pandemic. In this context, the present study is innovative, given the scarcity of previous investigations that assessed the effect of auriculotherapy on anxiety, quality of life and fear of COVID-19 in pregnant women treated in PHC. The relevance of the study for practice with the aforementioned population is also highlighted, since the intervention can impact health promotion, disease prevention, well-being and quality of life of pregnant women.

## OBJECTIVES

To assess the effect of auriculotherapy on anxiety in pregnant women treated in PHC and, secondarily, the effect of the intervention on the quality of life of these pregnant women and their fear of COVID-19.

## METHODS

### Study design, period and site

This is a randomized, single-blind (participant), multicenter, parallel, placebo-controlled clinical trial, divided into two groups in a 1:1 ratio, based on the CONsolidated Standards Of Reporting Trials (CONSORT)^(25)^ and STandards for Reporting Interventions in Clinical Trials of Acupuncture (STRICTA)^(26)^ guidelines. The study was developed in three cities located in northern Minas Gerais, Brazil, totaling 13 Basic Health Units (BHU), which assists a population of 33,281 people and 242 pregnant women, between February 2022 and July 2023. Regarding the composition of the BHU, all eight BHU of a small municipality were included, in addition to five random units, according to the geographic accessibility of two large municipalities.

### Population or sample; inclusion and exclusion criteria

Participants were pregnant women who received prenatal care at low-risk BHU and who met the study inclusion criteria. The following criteria were considered for inclusion in the study: (i) age over 18 years; (ii) gestational age up to 37 weeks, considering participation in all sessions and assessments; and (iii) moderate to high anxiety, assessed by a score ≥ 40 on the State-Trait Anxiety Inventory (STAI)^(27)^, cut-off point recognized in studies for clinically significant anxiety during pregnancy^(7,24)^.

The study excluded pregnant women who: (i) used benzodiazepines, antidepressants or anxiolytics; (ii) were undergoing treatment with integrative and complementary therapies or had undergone them in the last three months; (iii) had an infection, inflammation or injury to the ear; (iv) allergy to microporous tape; (v) use of piercing at the insertion site of the devices; and (vi) anatomical alteration in the ear. The criteria for discontinuation were: (i) absence in more than two consecutive sessions; (ii) deliveries during the study; and (iii) transfer to high-risk prenatal care.

For sample calculation, the difference in the mean anxiety scores, assessed using STAI, and initial and final assessments between the intervention group (IG) and the placebo group (PG) of the first 30 pregnant women included in the study were used using Repeated Measures with Attrition: Sample Sizes for 2 Groups (RMASS2^®^)^(28)^. On this occasion, a mean difference of 0.93 points in initial assessment and 1.8 points in final assessment was observed between groups.

A significance level of 5%, power of 80%, as in previous studies in a similar population^(24,29-31)^, and mean effect size of 0.5 were assumed, which corresponds to the size of the mean difference between groups (µ1-µ2/standard deviation) in standard deviation units. A sample of 51 pregnant women per group was estimated.

### Study protocol

In the first phase of the study, to recruit pregnant women, the study was publicized through posters and individual invitations made by nurses, community health workers and physicians from the participating BHU. Anxiety screening was carried out on the dates corresponding to prenatal appointments at the health units through STAI^(27)^. Pregnant women who presented STAI anxiety scores ≥40 and who met the selection criteria were invited after agreeing to participate in the study. At this stage, of the 195 pregnant women assessed for eligibility in the study, 76 were excluded because they obtained a score <40 on STAI, four withdrew from participation, one was using anxiolytics and one moved to another city. Then, the 113 participating pregnant women were randomized into IG and PG.

The randomization process was carried out in blocks of ten pregnant women, with a sequence of random numbers, obtained from the website *http://www.randomization.com/* by one of the researchers on the team, who did not participate in the data collection process. The list with the sequence of groups was placed in opaque, numbered and sealed envelopes. In initial assessment, the envelope was opened by the interventionist, immediately before the beginning of the intervention, to identify which group the pregnant women would belong to.

Four auriculotherapy sessions were performed once a week, with an average duration of 10 minutes. In IG, radionic crystals, fixed on microporous tape, were applied to the auricular points *Shen Men* (TF_4_), Kidney (CO_10_), Visceral Nervous System (AH_6_), Heart (CO_15_) and *Chuíqián* (LO_4_)^(32)^, previously identified in a literature review, followed by validation of appearance and content of the points protocol by 18 specialists with at least two years of academic and/or practical experience in the area of acupuncture and auriculotherapy and, finally, clinical validation. In PG, only the microporous tape was fixed, without any device, at the points equivalent to IG.

Prior to the application of auriculotherapy, the pinna was antisepsised with 70% alcohol. The radionic crystals and inert tapes were fixed unilaterally, with alternating sides at each session. The points were located using a point map from the World Federation of Acupuncture-Moxibustion Societies (WFAS)^(32)^. Manual stimulation of the points was not recommended due to the risk of injury to the pinna and the bias of the variable number of stimulations by pregnant women.

Application was carried out by two nurses and a dentist, who had an average of 19 months of experience in auriculotherapy. Uniformity and quality control of the site of auricular points was obtained through a four-hour training session on the protocol adopted in the study, with didactic instruction and supervised practice.

### Outcomes

In initial assessment, pregnant women answered the obstetric social and clinical characterization questionnaire, and instruments previously validated in Brazil, such as the State Anxiety Inventory (STAI)^(27)^, World Health Organization Quality of Life-Bref (WHOQOL-Bref)^(33)^ and Fear of COVID-19 Scale (FCV-19S)^(15)^. The second outcome assessment occurred in the last session, through the application of initial assessment instruments, in addition to the satisfaction form and the perception of the need for intervention by pregnant women. After seven days, the follow-up assessment was carried out with the application of STAI^(27)^, WHOQOL-Bref^(33)^ and FCV-19S^(15)^.

Anxiety was assessed using STAI. The inventory, created and validated in 1970 by Spielberg *et al*., translated and adapted to Brazilian Portuguese in 1979 by Biaggio and Natalício^(27)^, has been the most widely used during pregnancy due to the absence of specific instruments^(2,7)^ and because it presents reliability and validity in the Brazilian population^(27)^. The anxiety state subdivision has 20 four-point Likert-type questions and a score ranging from 20 to 80 points^(27)^. The higher the score, the greater the severity of the anxiety so that, in the context of pregnancy, the cut-off score is 40 points, which may indicate the need for clinical interventions due to the possibility of significant psychosomatic changes^(7,34,35)^. It should be noted that the clinical situation of the participating pregnant women did not influence adherence to the study and there was no need for further clinical management.

Quality of life was assessed using WHOQOL-Bref, an adapted version of WHOQOL-100, validated in 1998 and translated and validated in Brazil in 2000 by Fleck *et al*., with satisfactory psychometric characteristics^(36)^. WHOQOL-Bref assesses an individual’s physical and mental health through 26 five-point Likert-type questions across the physical, social, psychological and environmental domains, in addition to perceived quality of life and satisfaction with health^(33,37)^. Domain scores were calculated according to WHOQOL-Bref scoring manual^(33)^. Although WHOQOL-Bref does not have cut-off points, the higher the score, the higher the quality of life^(13)^.

To assess fear of COVID-19, FCV-19S was used. The translation and adaptation of FCV-19S into Brazilian Portuguese has nine items, with five-point Likert-type responses^(15)^. Scores range from nine to 45, and are considered reliable and valid for assessing the severity of fear of COVID-19 among women in the perinatal period in Brazil^(15)^. It is important to emphasize that the aforementioned scale was used mainly during the global public health emergency caused by COVID-19, and that its use outside this period may have variable results. Although there is no cut-off point for the classification of fear during pregnancy^(15)^, a stratification was adopted for the general public, in which little fear (<20 points), moderate fear (between 20 and 26 points) and very fear (>27 points) are considered^(38)^.

Social and clinical obstetric characterization was obtained through an instrument, in which information such as age, years of education, marital status, occupation, religion/belief, parity, types of previous births, planning and acceptance of current pregnancy, and number of prenatal appointments in current pregnancy were collected. The satisfaction of pregnant women with auriculotherapy was quantitatively assessed using an instrument developed by the authors, with Likert-type responses. On a scale of 1 to 5, participants indicated their degree of satisfaction with the intervention (“extremely dissatisfied”, “dissatisfied”, “not sure”, “satisfied”, “extremely satisfied”), the need for the intervention (“totally unnecessary”, “unnecessary”, “not sure”, “necessary”, “totally necessary”) and general health status after the end of treatment (“much better”, “better”, “no change”, “worse”, “much worse”). Moreover, the questionnaire included the symptoms or adverse effects associated with auriculotherapy at the end of treatment.

### Analysis of results, and statistics

Data were tabulated in Microsoft Office Excel^®^ version 365. Descriptive data analysis was performed in Statistical Package for the Social Sciences (SPSS) version 20 using simple frequency, measures of central tendency and measures of variability. Homogeneity and comparison of primary and secondary outcomes of IG and PG were analyzed using Generalized Estimating Equations (GEE), considering a 5% significance level. Nonparametric chi-square and Mann-Whitney tests, respectively, were performed to analyze the independent categorical and numerical covariates with non-normal distribution.

### Ethical considerations

The study was guided by Resolution 466/12 of the Ministry of Health regarding guidelines and standards for research involving human beings, and was approved by the Research Ethics Committee. Written informed consent was obtained from all individuals involved in the study. At the end of the study, pregnant women who received placebo auriculotherapy were invited to receive the same auriculotherapy protocol that IG received. Clinical trial was approved by the Brazilian Clinical Trials Registry (ReBEC), under identification RBR-7fsjmyc.

## RESULTS

**Figure 1:**
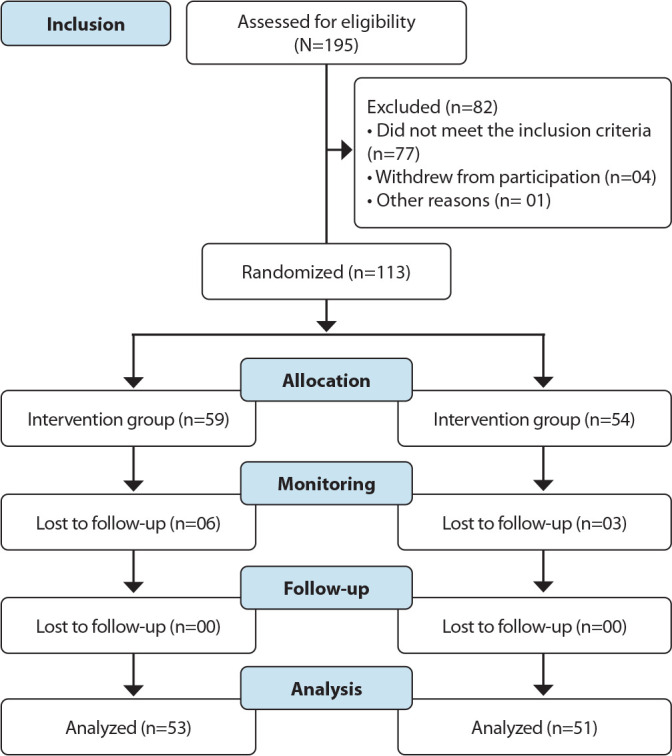
Sample screening and randomization flowchart adapted from CONsolidated Standards Of Reporting Trials, Montes Claros, Minas Gerais, Brazil, 2024

Among the 195 pregnant women screened, 82 were not eligible for randomization. Thus, 59 pregnant women were allocated to IG and 54 to PG. In the end, six pregnant women were lost to follow-up in IG and three in PG, leaving 53 participants in IG and 54 in PG (Figure 1).

Table 1 describes the comparison between groups in relation to sociodemographic, clinical and obstetric variables. Homogeneity between groups and adequacy in the randomization process were observed.

**Table 1:** Sample characterization in relation to sociodemographic, clinical and obstetric variables, Montes Claros, Minas Gerais, Brazil, 2024 (n=104)

Variables		IG (n=53)	PG (n=51)	*p* value
Age (m ± SD)	(years)	27.77±5.86	28.39±6.84	0.740^||^
Marital status (n, %)	Single	15 (28.30)	7 (13.73)	0.124^¶^
	Married or in a stable relationship	38 (71.70)	43 (84.31)	
	Divorced	0	1 (1.96)	
Years of study (m ± SD)	(years)	12.13±2.76	11.75±1.99	0.242^||^
Occupation (n, %)	Employed	20 (37.74)	17 (33.33)	0.357^¶^
	Unemployed	13 (24.53)	19 (37.25)	
	Self-employed	9 (16.98)	3 (5.88)	
	Student	1 (1.89)	1 (1.96)	
	Housewife	10 (18.87)	11 (21.57)	
Religious practice (n, %)	Practitioner	40 (75.47)	33 (64.71)	0.230^¶^
	Non-practitioner	13 (24.53)	18 (35.29)	
Religion/belief (n, %)	Catholic	36 (67.92)	35 (68.63)	0.325^¶^
	Evangelical	13 (24.53)	10 (19.61)	
	Atheist	2 (3.77)	5 (9.80)	
	Spiritualist	0 (0.00)	1 (1.96)	
	Other	2 (3.77)	0 (0.00)	
Previous diagnosis of anxiety (n, %)	Yes	22 (41.51)	18 (35.29)	0.515^¶^
	No	31 (58.49)	33 (64.71)	
Parity (n, %)	Primiparous	19 (35.85)	17 (33.33)	0.936^¶^
	Second-parous	18 (33.96)	17 (33.33)	
	Multiparous	16 (30.19)	17 (33.33)	
Previous childbirth route (n, %)	Vaginal	20 (37.74)	20 (39.22)	0.182^¶^
	Caesarian	8 (15.09)	12 (23.53)	
	Vaginal and caesarian	4 (7.55)	0 (0.00)	
	Not applicable	21 (39.62)	19 (37.25)	
Gestational age (n, %)	1st trimester	22 (41.51)	26 (50.98)	0.573^¶^
	2nd trimester	22 (41.51)	19 (37.25)	
	3rd trimester	9 (16.98)	6 (11.76)	
Pregnancy planning (n, %)	Yes	21 (39.62)	23 (45.10)	0.572^¶^
	No	32 (60.38)	28 (54.90)	
Acceptance of pregnancy (n, %)	Yes	48 (90.57)	49 (96.08)	0.262^¶^
	No	5 (9.43)	2 (3.92)	
Number of appointments (m ± SD)		3.94±2.37	3.51±2.49	0.228^||^

IG - intervention group; PG - placebo group; m - mean; SD - standard deviation;

|| Mann-Whitney;

¶ chi-square.

It was observed that there was no statistically significant difference in anxiety between groups in final assessment and follow-up (Table 2). However, over time, there was a statistically significant reduction in the mean obtained in STAI between initial and final assessments in IG (p=0.019) and initial and follow-up assessments in PG (p=0.001) (Table 2).

**Table 2:** Analysis of anxiety, verified by the State-Trait Anxiety Inventory, quality of life, verified by the World Health Organization Quality of Life-Bref, and perception of fear of COVID-19, verified by the Fear of COVID-19 Scale, expressed as mean, standard error and 95% Confidence Interval, according to the Generalized Estimating Equations model, Montes Claros, Minas Gerais, Brazil, 2024 (n=104)

Outcome variable	Group	Initial	Final	Follow-up	*p* value		
		m(se)	m(se)	m(se)	Initial-final	Final-follow-up	Initial-follow-up
STAI	IG	50.36(0.63) ^‡§^	47.92(0.96)^[Table-fn TFN5] ‡§^	48.00(0.94)^[Table-fn TFN5] ‡§^	0.019^||^	1.000	0.073
PG	50.82(0.56)^[Table-fn TFN5] ‡§^	49.04(0.82)^[Table-fn TFN5] ‡§^	48.49(0.88)^[Table-fn TFN5] ‡§^	0.05	0.841	**0.001^||^**
*p* value	0.581	0.377	0.703			
WHOQOL-perceived quality of life	IG	71.23(2.53)^[Table-fn TFN5] ‡§^	77.83(2.37)^[Table-fn TFN5] ‡§^	75.94(2.40)^[Table-fn TFN5] ‡§^	**0.041^||^**	0.281	0.233
	PG	72.06(1.79)^[Table-fn TFN5] ‡§^	75.49(1.90)^[Table-fn TFN5] ‡§^	74.02(1.83)^[Table-fn TFN5] ‡§^	0.185	0.752	0.938
	*p* value	0.788	0.441	0.524			
WHOQOL-satisfaction with health	IG	69.23(3.09)^[Table-fn TFN5] ‡§^	75.00(2.31)^[Table-fn TFN5] ‡§^	73.11(2.48)^[Table-fn TFN5] ‡§^	0.106	0.938	0.726
	PG	69.00(2.96)^[Table-fn TFN5] ‡§^	71.08(2.81)^[Table-fn TFN5] ‡§^	71.77(2.50)^[Table-fn TFN5] ‡§^	1.000	1.000	0.829
	*p* value	0.957	0.281	0.661			
WHOQOL-physical domain	IG	59.57(2.11)^[Table-fn TFN5] ‡§^	70.82(1.79)^[Table-fn TFN5] ‡§^	70.22(1.86)^[Table-fn TFN5] ‡§^	**<0.001^||^**	1.000	**<0.001^||^**
	PG	62.25(2.29)^[Table-fn TFN5] ‡§^	63.59(2.29)^[Table-fn TFN5] ‡§^	63.94(2.30)^[Table-fn TFN5] ‡§^	1.000	1.000	0.761
	*p* value	0.389	**0.013^||^**	**0.034^||^**			
WHOQOL-psychological domain	IG	59.83(2.57)^[Table-fn TFN5] ‡§^	67.45(2.14)^[Table-fn TFN5] ‡§^	66.43(2.04)^[Table-fn TFN5] ‡§^	**<0.001^||^**	1.000	**0.005^||^**
	PG	57.76(2.20)^[Table-fn TFN5] ‡§^	62.50(2.40)^[Table-fn TFN5] ‡§^	61.76(2.41)^[Table-fn TFN5] ‡§^	**0.016^||^**	1.000	**0.019^||^**
	*p* value	0.542	0.123	0.139			
WHOQOL- social domain	IG	66.03(2.25)^[Table-fn TFN5] ‡§^	69.65(2.36)^[Table-fn TFN5] ‡§^	69.03(2.38)^[Table-fn TFN5] ‡§^	0.302	1.000	0.554
	PG	65.50(2.40)^[Table-fn TFN5] ‡§^	68.17(2.78)^[Table-fn TFN5] ‡§^	69.33(2.65)^[Table-fn TFN5] ‡§^	0.726	1.000	0.162
	*p* value	0.873	0.683	0.931			
WHOQOL-environmental domain	IG	61.50(1.83)^[Table-fn TFN5] ‡§^	65.50(1.69)^[Table-fn TFN5] ‡§^	65.80(1.69)^[Table-fn TFN5] ‡§^	**0.010^||^**	1.000	**0.008^||^**
	PG	57.91(1.84)^[Table-fn TFN5] ‡§^	58.95(1.96)^[Table-fn TFN5] ‡§^	58.10(2.13)^[Table-fn TFN5] ‡§^	1.000	1.000	1.000
	*p* value	0.166	**0.011^||^**	**0.005^||^**			
FCV-19S	IG	29.15(0.89)^[Table-fn TFN5] ‡§^	26.47(0.95)^[Table-fn TFN5] ‡§^	26.00(0.92)^[Table-fn TFN5] ‡§^	**<0.001^||^**	0.331	**<0.001^||^**
	PG	28.31(0.82)^[Table-fn TFN5] ‡§^	26.43(0.83)^[Table-fn TFN5] ‡§^	26.41(0.85)^[Table-fn TFN5] ‡§^	**<0.001^||^**	1.000	**<0.001^||^**
	*p* value	0.489	0.975	0.742			

IG - intervention group; PG - placebo group;

‡ m - mean;

§ se - standard error;

|| p<0.005; WHOQOL-Bref - World Health Organization Quality of Life-Bref; FCV-19S - Fear of COVID-19 Scale; STAI - State-Trait Anxiety Inventory.

Concerning perceived quality of life, there was no statistically significant difference in the mean between groups at the three assessment times. However, there was a statistically significant improvement in the mean of IG between initial and final assessments (p= 0.041). In terms of satisfaction with health, there was no statistically significant difference in the mean between IG and PG at final assessment and follow-up, and there was no difference in the mean over time within each group (Table 2).

In the physical domain of WHOQOL-Bref, there was a statistically significant difference between groups in final assessment (p<0.001) and follow-up (p<0.001) so that IG presented a better score in this domain in relation to PG (mean difference: 7.23). Over time, there was a statistically significant improvement between initial and final assessments (p=0.013) and initial and follow-up (p=0.034) only in IG (Table 2).

In the psychological domain of WHOQOL-Bref, there was no statistically significant difference between groups in final assessment and follow-up (Table 2). However, there was a statistically significant improvement between initial and final assessments in IG (p<0.001) and in PG (p=0.016) and between initial and follow-up assessments in IG (p=0.005) and in PG (p=0.019).

In the social domain of WHOQOL-Bref, there was no statistically significant difference in the mean between treatment groups over time and between groups. In the environmental domain of WHOQOL-Bref, there was a statistically significant difference in the mean between the treatment groups at final assessment (p=0.011) and follow-up (p=0.005) so that IG presented better scores in this domain in relation to PG at the final moment (mean difference: 6.55) and at follow-up (mean difference: 7.7). Furthermore, there was a statistically significant improvement in this domain between initial and final assessments (p=0.010) and initial and follow-up assessments (p=0.008) in IG (Table 2).

As for fear of COVID-19, assessed by FCV-19S, throughout the treatment, there was no statistically significant difference between IG and PG in final assessment and follow-up. However, there was a statistical reduction in the mean fear of COVID-19 between initial and final assessments in IG (p<0.001) and PG (p<0.001) and between initial and follow-up assessments in IG (p<0.001) and PG (p<0.001) (Table 2).

Most pregnant women were satisfied or extremely satisfied with the intervention, regardless of the group. Furthermore, most pregnant women in IG considered the intervention necessary or completely necessary. Among PG participants, there were women who were unsure about the need for the intervention and who considered it necessary or completely necessary. Most pregnant women in IG considered their general condition better or much better. Pregnant women in PG reported that their general condition was better or had no change (Table 3).

**Table 3:** Satisfaction of pregnant women regarding treatment and the need for intervention, Montes Claros, Minas Gerais, Brazil, 2024 (n=104)

Variables		IG (n=53)	PG (n=51)
Satisfaction with the intervention performed (n, %)	Not sure	3 (5.66)	10 (19.61)
	Satisfied	34 (64.15)	33 (64.71)
	Extremely satisfied	16 (30.19)	8 (15.69)
Perception of the need for intervention (n, %)	Not sure	1 (1.89)	14 (27.45)
	Necessary	39 (73.58)	26 (50.98)
	Totally necessary	13 (24.53)	11 (21.57)
General condition after treatment (n, %)	Much better	15 (28.30)	7 (13.73)
	Better	36 (67.92)	33 (64.71)
	No change	2 (3.77)	11 (21.57)

*IG - intervention group; PG - placebo group.*

Most pregnant women did not report significant adverse reactions, demonstrating the safety of the auriculotherapy intervention. Of the total number of pregnant women assessed, 86.54% (n=90) did not present any symptoms or adverse reactions. The remaining women who presented symptoms had headache related to fixation in the pinna in IG, pain in the pinna in IG and PG, and itching in the pinna in both groups (Table 4).

**Table 4:** Frequency and intensity of symptoms or adverse reactions in people who received auricular acupuncture, Montes Claros, Minas Gerais, Brazil, 2024 (n=14)

Symptoms or adverse reactions (n=14)	IG		PG	
	Frequency n (%)	Intensity (m±DP)	Frequency n (%)	Intensity (m±DP)
Headache related to pinna fixation	2 (3.77)	3.50±3.54	0 (0.00)	0.00±0.00
Pain in the pinna	5 (9.43)	4.40±1.95	1 (1.96)	2.00±0.00
Itching in the pinna	2 (3.77)	1.50±0.71	4 (7.84)	6.00±3.74

*IG - intervention group; PG - placebo group; m - mean; SD - standard deviation.*

## DISCUSSION

Auriculotherapy demonstrated a positive and similar effect on anxiety in both IG and PG. However, in IG, superior effectiveness was identified in the physical and environmental domains of quality of life. Furthermore, the groups presented similar and positive effects in the psychological domain of quality of life and in fear of COVID-19, with no statistical difference between groups. In the social domain and in perceived quality of life, there was no statistical difference in both groups. Satisfaction with health was greater with auriculotherapy between initial and final assessments, even though no statistical difference was noted between groups.

The present study did not demonstrate a statistically significant difference between groups for anxiety. However, in a Brazilian clinical trial, in which the effect of auriculotherapy was assessed compared to a control group with regular nursing appointments in 50 low-risk pregnant women undergoing prenatal care, there was a statistically significant difference^(24)^. The superior effect of auriculotherapy on PG and the control group was also observed in a clinical trial with 102 postpartum women with anxiety^(28)^ and a study with 150 women who underwent uterine aspiration in the first trimester^(29)^. It is important to note that the population and the setting of this study have differences in the gestational period that influence the results.

Thus, it was observed that auriculotherapy has a superior effect to control groups with usual care, despite there being no statistical difference with PGs in situations of abortion^(30,39)^ and usual-risk prenatal care, as found in the present study. However, it is emphasized that listening and addressing emotional aspects carried out by nurses demonstrated a therapeutic effect in PG. Reduced anxiety in PG can also be justified by the acceptability and satisfaction with the technique^(40)^ and by the psychological mechanisms that result in the relief of anxiety, through conditioning and expectation for treatment in PG^(41)^.

Auriculotherapy demonstrated a significant effect on the physical domain of quality of life, as well as the results of a clinical trial carried out in Spain, which assessed the effect of auriculotherapy associated with standard obstetric care in primary care, finding an increase of 40.5% in this domain in the auriculotherapy group, compared to an increase of 8.1% in the control group^(42)^.

In relation to the psychological domain of quality of life, the increase in the averages in IG and PG demonstrated that the approach to anxiety, quality of life and fears that permeate pregnancy positively influences this domain^(40)^. In the literature, among the approaches with integrative and complementary practices, spirituality has been assessed as an important component of psychological well-being^(23,43)^. However, no other studies were found that assessed the effect of auriculotherapy specifically in this domain.

It is believed that the social domain did not undergo variations because it was associated with factors external to therapy, which include social relationships, care, love, comfort, sexuality, good marital, family and friend relationships^(44)^. In this context, it is worth highlighting that social support for pregnant women has a positive correlation with quality of life, and is considered a protective factor for anxiety^(45)^ and fear of COVID-19^(12)^.

The environment domain, which assesses financial resources, availability and quality of social and health care, showed a statistically significant difference for the auriculotherapy group. In the literature, it was observed that the economic issue is one of the main negative factors in quality of life^(46,47)^ and anxiety^(7,48)^. However, it is possible to note that health care is capable of improving the scores in this domain, given that pregnant women in the auriculotherapy group demonstrated greater satisfaction and higher rates of perception that the intervention was necessary or completely necessary.

In the national and international literature, there is a lack of evidence regarding the impact of ICHP on fear of COVID-19. However, in the general population of Turkey, 39.3% used these practices during the pandemic, with acupuncture used by 3.7% of these people^(20)^. In the present study, in IG and PG, fear of COVID-19 reduced from very to moderate, reinforcing that listening to and addressing fear positively impacts the condition^(40)^.

Regarding adverse events of auriculotherapy, headache, pain and itching in the pinna with mild to moderate intensity were observed. Maternal and fetal outcomes with the use of auriculotherapy were not reported as well as a clinical trial that assessed the effect of auriculotherapy on low back pain in pregnant women^(42)^. Thus, this and other evidence has demonstrated that, like systemic acupuncture^(49)^, auriculotherapy is safe for both the mother and the fetus^(24,42)^.

### Study limitations

Among the limitations of this study are the socioeconomic and cultural particularities of the population studied, observed in the homogeneity of the clinical, obstetric and sociodemographic characterization of pregnant women, which makes the results not generalizable to other realities. In addition to this, although STAI is widely used in studies with pregnant women, the absence of a translated and validated instrument for assessing anxiety related to pregnancy may have influenced the results of this variable in the research.

### Contributions to nursing, health or public policy

This study contributed to science and nursing, as it demonstrated the safety of auriculotherapy for anxiety during pregnancy, using a non-invasive device. Additionally, most pregnant women, regardless of the allocation group, considered themselves satisfied or very satisfied and that the treatment was necessary or totally necessary, probably due to the lack of a mental health approach during prenatal care and the focus on the physical aspects of pregnancy. These findings reinforce the need for nurses and multidisciplinary teams to screen conditions such as anxiety, quality of life and fear of COVID-19, and implement prevention and control measures such as auriculotherapy.

Therefore, it is believed that this study may contribute to the care of low-risk pregnant women followed in PHC, in addition to being essential for future studies in different contexts and sample sizes. Furthermore, it contributes to the clinical judgment about anxiety in this population and, consequently, to the impact of higher quality nursing care based on strong scientific evidence. Therefore, it is recommended that supplies be acquired and professionals working in PHC be trained to use the technique as an instrument capable of helping to reduce anxiety and improve pregnant women’s quality of life.

## CONCLUSIONS

Auriculotherapy demonstrated a similar effect to PG for anxiety treatment in low-risk pregnant women. However, it demonstrated a superior effect to PG in the physical and environmental domains of quality of life. The impact of the effect on these domains of quality of life may contribute to the advancement of knowledge in health and nursing about the effects of auriculotherapy in this population and context. Furthermore, the technique proved to be safe, with no serious adverse events reported by the pregnant women who participated in the study. Finally, the results indicate that the auriculotherapy protocol adopted may be beneficial in clinical practice, and future investigations in different contexts are recommended.
