# Supplementary material for: Auriculotherapy for anxiety, quality of life and fear of COVID-19 in pregnant women: a randomized clinical trial
Source: Rev Bras Enferm. 2025 Apr 28;78(2):e20240062. doi: 10.1590/0034-7167-2024-0062 (PMC12037191; doi:10.1590/0034-7167-2024-0062)
Supplement: Translation

# Translation: Auriculoterapia para ansiedade, qualidade de vida e medo da COVID-19 em gestantes: ensaio clínico randomizado

**DOI:** 10.1590/0034-7167-2024-0062pt

**Authors:** Hérica Pinheiro Corrêa, Kauê Batista Andrade, Tânia Couto Machado Chianca, Antônio Prates Caldeira, Carolina Amaral Oliveira Rodrigues, Maria Fernanda Santos Figueiredo Brito, Caroline de Castro Moura, Diego Dias de Araújo

**Affiliations:** I Universidade Estadual de Montes Claros. Montes Claros, Minas Gerais, Brasil; II Universidade Federal de Minas Gerais. Belo Horizonte, Minas Gerais, Brasil; III Universidade Federal de Viçosa. Viçosa, Minas Gerais, Brasil

**Keywords:** Auriculoterapia, Ansiedade, Qualidade de Vida, COVID-19, Ensaio Clínico

## Abstract

**Objetivos:**

avaliar o efeito da auriculoterapia sobre ansiedade, qualidade de vida e medo da COVID-19 em gestantes.

**Métodos:**

ensaio clínico randomizado multicêntrico, simples-cego. Participaram 53 gestantes no grupo intervenção e 51 no placebo. Os desfechos foram mensurados pelo Inventário de Ansiedade Estado, *World Health Organization Quality of Life-Bref*, Escala do Medo da COVID-19 Perinatal e avaliação da intervenção. Os dados foram analisados pelo *Generalized Estimating Equations*.

**Resultados:**

em ambos os grupos, houve redução da ansiedade. Na qualidade de vida, o grupo intervenção apresentou melhora significativa nos domínios físico e ambiental. No domínio psicológico, houve melhora em ambos os grupos, e no domínio social, não houve alteração significativa. O medo da COVID-19 diminuiu significativamente em ambos os grupos.

**Conclusões:**

a auriculoterapia apresentou efeito igual ao do grupo placebo na redução da ansiedade e no medo da COVID-19, e efeito superior nos domínios físico e ambiental da qualidade de vida. REBEC: RBR-7fsjmyc.

## INTRODUÇÃO

A gravidez predispõe e torna a mulher mais exposta à ansiedade pelas alterações fisiológicas, psicossociais, preocupações atuais e futuras em relação à gestação^(1)^. A ansiedade relacionada à gravidez se diferencia dos transtornos de ansiedade geral, pois é específica aos desfechos da gravidez, parto, pós-parto, saúde do bebê e materna^(2)^.

A prevalência de ansiedade na gestação, descrita na literatura mundial, é de 18,2% no primeiro trimestre, aumentando para 19,1% no segundo trimestre e 24,6% no terceiro trimestre^(3)^. No Brasil, em estudo multicêntrico transversal, observou-se que, durante a pandemia de COVID-19, 16,1% gestantes apresentaram ansiedade materna moderada e, 11,5%, ansiedade materna grave^(4)^.

A ansiedade é uma condição multifatorial, e sua interação com o sistema imunológico, neuroendócrino e placenta tem impacto significativo na saúde e no bem-estar materno-fetal, com consequências a curto e longo prazo^(5-8)^. Os impactos negativos incluem risco aumentado de desfechos obstétricos negativos^(9)^, tais como crescimento intrauterino restrito^(10)^, baixo peso ao nascer, aborto espontâneo e parto prematuro^(11)^.

No contexto pandêmico, o medo do desconhecido, associado à espera de um bebê, acesso à informação e maior morbidade e mortalidade pelo vírus em mulheres grávidas gerou adoecimento mental^(12)^ e impacto negativo na qualidade de vida das gestantes^(13,14)^. Dessa forma, o medo crônico ou inapropriado, que está intimamente relacionado a transtornos mentais comuns, como ansiedade, foi alvo de monitoramento e intervenções^(13,15)^, além de investigações, como o presente estudo.

O manejo das alterações emocionais e psicológicas, entre as quais se destaca a ansiedade, é realizado na Atenção Primária à Saúde (APS)^(16)^. Porém, o tratamento dessa condição recebe pouca atenção dos profissionais e das gestantes, devido à percepção de que os sintomas da ansiedade, mesmo quando patológica, são naturais da gravidez e pela falta de abordagem dos aspectos psicossociais^(17)^.

Nesse contexto, o enfermeiro se destaca, tendo em vista que, após a identificação das alterações emocionais e psicológicas, como a ansiedade na gestação, planeja e implementa estratégias de enfrentamento adaptativo^(17)^. Entre as intervenções, as Práticas Integrativas e Complementares em Saúde (PICS), em especial auriculoterapia, têm sido amplamente utilizadas por enfermeiros para condução de situações emocionais^(18,19)^.

No cenário global, a utilização das PICS pela população em geral varia entre 39,3%^(20)^ e 51,8%^(21)^, e estima-se que uma a cada três gestantes as utilizam^(22)^. Em muitas realidades, as intervenções não farmacológicas são a primeira opção de tratamento para ansiedade^(10)^. Uma vez que as PICS têm sido consideradas estratégias de promoção da saúde e de tratamento de queixas comuns na gestação^(22,23)^, é importante que os profissionais de saúde tenham conhecimento sobre as intervenções, considerando a possibilidade da influência dessas na gravidez e as possíveis interações com medicamentos e complicações fetais^(22)^.

Contudo, apesar da eficácia e segurança da auriculoterapia para ansiedade na população em geral^(18)^, há carência de estudos acerca dessa intervenção em gestantes no período pré-natal^(24)^, bem como daqueles desenhados para avaliar o medo da COVID-19 e qualidade de vida de gestantes no contexto da pandemia de COVID-19. Nesse contexto, o presente estudo possui caráter inovador, visto a escassez de investigações prévias que avaliaram o efeito da auriculoterapia sobre ansiedade, qualidade de vida e medo da COVID-19 em gestantes atendidas na APS. Destaca-se também a relevância do estudo para prática junto à população citada, uma vez que a intervenção pode impactar a promoção saúde, a prevenção de agravos, o bem-estar e a qualidade de vida das gestantes.

## OBJETIVOS

Avaliar o efeito da auriculoterapia sobre a ansiedade em gestantes atendidas na APS e, secundariamente, o efeito da intervenção sobre a qualidade de vida dessas gestantes e o medo da COVID-19.

## MÉTODOS

### Desenho, período e local do estudo

Ensaio clínico randomizado, simples-cego (participante), multicêntrico, paralelo e controlado por placebo, dividido em dois grupos na proporção 1:1, pautado nas diretrizes do *CONsolidated Standards Of Reporting Trials* (CONSORT)^(25)^ e do *STandards for Reporting Interventions in Clinical Trials of Acupuncture* (STRICTA)^(26)^. O estudo foi desenvolvido em três cidades localizadas no norte de Minas Gerais, Brasil, totalizando 13 Unidades Básicas de Saúde (UBS), que atendem uma população de 33.281 pessoas e 242 gestantes, entre fevereiro de 2022 e julho de 2023. Quanto à composição das UBS, todas as oito UBS de um município de pequeno porte foram incluídas, além de cinco unidades aleatórias, conforme acessibilidade geográfica de dois municípios de grande porte.

### População ou amostra; critérios de inclusão e exclusão

As participantes foram gestantes que fizeram acompanhamento de pré-natal de risco habitual nas UBS e que atenderam aos critérios de inclusão do estudo. Para inclusão das gestantes no estudo, foram considerados os seguintes critérios: (i) idade maior que 18 anos; (ii) idade gestacional até 37 semanas, tendo em vista a participação em todas as sessões e avaliações; e (iii) ansiedade moderada a alta, avaliada pelo escore ≥ 40 no Inventário de Ansiedade Traço-Estado (IDATE-E)^(27)^, ponto de corte reconhecido nos estudos para o estado de ansiedade clinicamente significativo na gestação^(7,24)^.

Foram excluídas do estudo as gestantes que: (i) utilizavam medicamentos benzodiazepínicos, antidepressivos ou ansiolíticos; (ii) estivessem em tratamento com terapias integrativas e complementares ou as realizou nos últimos três meses; (iii) infecção, inflamação ou ferimento no pavilhão auricular; (iv) alergia a fita microporosa; (v) uso de *piercing* no local de inserção dos dispositivos; e (vi) alteração anatômica no pavilhão auricular. Os critérios de descontinuidade foram: (i) falta em mais de duas sessões consecutivas; (ii) partos durante a realização do estudo; e (iii) transferência para o pré-natal de alto risco.

Para o cálculo amostral, foi utilizada a diferença das médias da ansiedade, avaliada por meio do IDATE-E, e da avaliação inicial e final entre o grupo intervenção (GI) e o grupo placebo (GP) das primeiras 30 gestantes incluídas no estudo, por meio do *software Repeated Measures with Attrition: Sample Sizes for 2 Groups* (RMASS2^®^)^(28)^. Nessa ocasião, foi observada diferença de média de 0,93 pontos na avaliação inicial e de 1,8 pontos na avaliação final entre os grupos.

Foram assumidos o nível de significância de 5%, poder de 80%, conforme estudos prévios em população similar^(24,29-31)^, e tamanho de efeito médio de 0,5, que corresponde ao tamanho da diferença média entre os grupos (µ1-µ2/desvio padrão) em unidades de desvio padrão. Foi estimada uma amostra de 51 gestantes por grupo.

### Protocolo do estudo

Na primeira fase do estudo, para recrutamento das gestantes, o estudo foi divulgado por meio de cartazes e convite individual realizado pelos enfermeiros, agentes comunitários de saúde e médicos das UBS participantes. Já o rastreamento da ansiedade foi realizado nas datas correspondentes às consultas de pré-natal nas unidades de saúde por intermédio do IDATE-E^(27)^. As gestantes que apresentaram escores de ansiedade no IDATE-E ≥40 e que atenderam aos critérios de seleção foram convidadas, após aceitarem participar da pesquisa. Nessa etapa, das 195 gestantes avaliadas para elegibilidade no estudo, 76 foram excluídas, pois obtiveram escore <40 no IDATE-E, quatro desistiram de participar, uma fazia uso de ansiolítico e uma mudou de cidade. Em seguida, as 113 gestantes participantes foram aleatorizadas no GI e no GP.

O processo de randomização foi realizado em blocos de dez gestantes, com uma sequência de números aleatórios, obtidos no *site* http://www.randomization.com/ por um dos pesquisadores da equipe, que não participou do processo de coleta de dados. A lista com a sequência dos grupos foi colocada em envelopes opacos, numerados e lacrados. Na avaliação inicial, o envelope foi aberto pelo intervencionista, imediatamente antes do início da intervenção, para se identificar a que grupo a gestante pertenceria.

Foram realizadas quatro sessões de auriculoterapia, uma vez por semana, com tempo médio de 10 minutos. No GI, os cristais radiônicos, fixados em fita microporosa, foram aplicados nos pontos auriculares *Shen Men* (TF_4_), Rim (CO_10_), Sistema Nervoso Visceral (AH_6_), Coração (CO_15_) e *Chuíqián* (LO_4_)^(32)^, identificados previamente em revisão de literatura, seguida de validação de aparência e conteúdo do protocolo de pontos por 18 especialistas com experiência acadêmica e/ou prática mínima de dois anos na área da acupuntura e auriculoterapia e, por fim, validação clínica. No GP, foi fixada apenas a fita microporosa, sem nenhum dispositivo, nos pontos equivalentes ao GI.

Anteriormente à aplicação da auriculoterapia, foi realizada antissepsia do pavilhão auricular com álcool 70%. Os cristais radiônicos e fitas inertes foram fixados de maneira unilateral, com alternância de lado a cada sessão. A localização dos pontos foi por meio de mapa de pontos da *World Federation of Acupuncture-Moxibustion Societies* (WFAS)^(32)^. Não foi orientada estimulação manual dos pontos, devido ao risco de lesão no pavilhão auricular e viés de número de estimulação variável pelas gestantes.

A aplicação foi realizada por duas enfermeiras e um dentista, que possuíam experiência média de 19 meses na realização da prática de auriculoterapia. O controle da uniformidade e qualidade da localização dos pontos auriculares foi obtida por meio de capacitação de quatro horas acerca do protocolo adotado no estudo, com instrução didática e prática supervisionada.

### Desfechos

Na avaliação inicial, as gestantes responderam ao questionário de caracterização social e clínica obstétrica, e aos instrumentos, previamente validados no Brasil, como Inventário de Ansiedade Estado (IDATE-E)^(27)^, *World Health Organization Quality of Life-Bref* (WHOQOL-Bref)^(33)^ e Escala do Medo da COVID-19 Perinatal (EMC19-9)^(15)^. A segunda avaliação de desfecho ocorreu na última sessão, por meio da aplicação dos instrumentos da avaliação inicial, acrescidos do formulário de satisfação e da percepção da necessidade da intervenção pela gestante. Após sete dias, foi realizada a avaliação de seguimento/*follow-up* com a aplicação do IDATE-E^(27)^, WHOQOL-Bref^(33)^ e EMC19-9^(15)^.

A ansiedade foi avaliada por meio do IDATE-E. O inventário, criado e validado em 1970 por Spielberg *et al.*, traduzido e adaptado para o português brasileiro em 1979 por Biaggio e Natalício^(27)^, tem sido o mais utilizado na gestação pela ausência de instrumentos específicos^(2,7)^ e por apresentar fidedignidade e validade na população brasileira^(27)^. A subdivisão do estado de ansiedade tem 20 questões do tipo Likert de quatro pontos e escore que varia de 20 a 80 pontos^(27)^. Quanto maior o escore, maior a gravidade da ansiedade, de modo que, no contexto da gestação, a nota de corte é de 40 pontos, que pode indicar a necessidade de intervenções clínicas devido à possibilidade de alterações psicossomáticas significativas^(7,34,35)^. Ressalta-se que a situação clínica das gestantes participantes não influenciou a adesão ao estudo e não houve necessidade de maiores manejos clínicos.

A qualidade de vida foi avaliada por meio do WHOQOL-Bref, uma versão adaptada do WHOQOL-100, validado em 1998 e traduzido e validado no Brasil em 2000 por Fleck *et al.*, com características psicométricas satisfatórias^(36)^. O WHOQOL-Bref avalia a saúde física e mental do indivíduo, por meio de 26 questões do tipo Likert de cinco pontos, através dos domínios físico, social, psicológico e do meio ambiente, além da percepção da qualidade de vida e da satisfação com a saúde^(33,37)^. As pontuações dos domínios foram calculadas de acordo com o manual de pontuação do WHOQOL-Bref^(33)^. Embora o WHOQOL-Bref não tenha pontos de corte, quanto maior o escore, maior a qualidade vida^(13)^.

Para avaliação do medo da COVID-19, utilizou-se a EMC19-9. A tradução e adaptação da EMC19-9 para o português do Brasil possui nove itens, com respostas do tipo Likert de cinco pontos^(15)^. As notas variam de nove a 45, e são consideradas confiáveis e válidas para avaliar a gravidade do medo da COVID-19 entre mulheres em período perinatal no Brasil^(15)^. Enfatiza-se que a escala supracitada foi utilizada, principalmente, durante a emergência de saúde pública global causada pela COVID-19, e que o uso fora desse período pode ter resultados variáveis. Embora não exista ponto de corte para a classificação do medo na gestação^(15)^, foi adotada a estratificação para o público em geral, na qual se consideram pouco medo (<20 pontos), medo moderado (entre 20 e 26 pontos) e muito medo (>27 pontos)^(38)^.

A caracterização social e clínica obstétrica foi obtida por um instrumento, no qual foram colhidas informações como idade, anos de estudo, estado civil, ocupação, religião/crença, paridade, tipos de partos anteriores, planejamento e aceitação da gestação atual, e número de consultas de pré-natal na gestação atual. A satisfação das gestantes com a auriculoterapia foi avaliada quantitativamente por meio de um instrumento, elaborado pelos autores, com respostas do tipo Likert. Em uma escala de 1 a 5, as participantes apontaram o grau de satisfação com a realização da intervenção (“extremamente insatisfeito”, “insatisfeito”, “não tem certeza”, “satisfeito”, “extremamente satisfeito”), a necessidade de realização da intervenção (“totalmente desnecessária”, “desnecessária”, “não tem certeza”, “necessária”, “totalmente necessária”) e o estado geral de saúde após o término do tratamento (“muito melhor”, “melhor”, “nenhuma mudança”, “pior”, “muito pior”). Além disso, o questionário englobava os sintomas ou efeitos adversos associados à auriculoterapia no final do tratamento.

### Análise dos resultados e estatística

Os dados foram tabulados no *software Microsoft Office Excel* ^®^, versão 365. A análise descritiva dos dados foi realizada no *software Statistical Package for the Social Sciences* (SPSS), versão 20, por meio de frequência simples, medidas de tendência central e medidas de variabilidade. A homogeneidade e a comparação dos desfechos primário e secundários do GI e GP foram analisadas pelo *Generalized Estimating Equations* (GEE), considerando 5% de significância. Os testes não paramétricos do qui-quadrado e Mann-Whitney, respectivamente, foram realizados para análise das covariáveis independentes categóricas e numéricas com distribuição não normal.

### Considerações éticas

O estudo foi pautado na Resolução n^o^ 466/12 do Ministério da Saúde acerca das diretrizes e normas de pesquisas que envolvem seres humanos, sendo aprovado por Comitê de Ética em Pesquisa. O Termo de Consentimento Livre e Esclarecido foi obtido de todos os indivíduos envolvidos no estudo por meio escrito. Ao final do estudo, as gestantes que receberam auriculoterapia placebo foram convidadas a receber o mesmo protocolo de auriculoterapia que o GI recebeu. O ensaio clínico foi homologado no Registro Brasileiro de Ensaios Clínicos (ReBEC), sob identificação RBR-7fsjmyc.

## RESULTADOS

Entre as 195 gestantes do rastreamento, 82 não foram elegíveis para randomização. Assim, 59 gestantes foram alocadas no GI, e 54, no GP. Ao final, seis gestantes foram perdidas no acompanhamento do GI, e três, no GP, restando 53 participantes no GI e 54 no GP (Figura 1).

**Figura 1:** Fluxograma de rastreamento e randomização da amostra adaptado do *CONsolidated Standards Of Reporting Trials*, Montes Claros, Minas Gerais, Brasil, 2024

A comparação entre os grupos em relação às variáveis sociodemográficas, clínicas e obstétricas está descrita na Tabela 1. Constataram-se a homogeneidade entre os grupos e a adequação no processo de randomização.

**Tabela 1:** Caracterização da amostra em relação às variáveis sociodemográficas, clínicas e obstétricas, Montes Claros, Minas Gerais, Brasil, 2024 (n=104)

Variáveis		GI (n=53)	GP (n=51)	Valor de *p*
Idade (m ± DP)	(anos)	27,77±5,86	28,39±6,84	0,740^||^
Estado civil (n, %)	Solteira	15 (28,30)	7 (13,73)	0,124^¶^
	Casada ou união estável	38 (71,70)	43 (84,31)	
	Divorciada	0	1 (1,96)	
Anos de estudo (m ± DP)	(anos)	12,13±2,76	11,75±1,99	0,242^||^
Ocupação (n, %)	Empregada	20 (37,74)	17 (33,33)	0,357^¶^
	Desempregada	13 (24,53)	19 (37,25)	
	Autônoma	9 (16,98)	3 (5,88)	
	Estudante	1 (1,89)	1 (1,96)	
	Do lar	10 (18,87)	11 (21,57)	
Prática religiosa (n, %)	Praticante	40 (75,47)	33 (64,71)	0,230^¶^
	Não praticante	13 (24,53)	18 (35,29)	
Religião/crença (n, %)	Católica	36 (67,92)	35 (68,63)	0,325^¶^
	Evangélica	13 (24,53)	10 (19,61)	
	Ateia	2 (3,77)	5 (9,80)	
	Espiritualista	0 (0,00)	1 (1,96)	
	Outra	2 (3,77)	0 (0,00)	
Diagnóstico prévio de ansiedade (n, %)	Sim	22 (41,51)	18 (35,29)	0,515^¶^
	Não	31 (58,49)	33 (64,71)	
Paridade (n, %)	Primípara	19 (35,85)	17 (33,33)	0,936^¶^
	Secundípara	18 (33,96)	17 (33,33)	
	Multípara	16 (30,19)	17 (33,33)	
Via de parto anterior (n, %)	Vaginal	20 (37,74)	20 (39,22)	0,182^¶^
	Cesariana	8 (15,09)	12 (23,53)	
	Vaginal e cesariana	4 (7,55)	0 (0,00)	
	Não se aplica	21 (39,62)	19 (37,25)	
Idade gestacional (n, %)	1º trimestre	22 (41,51)	26 (50,98)	0,573^¶^
	2º trimestre	22 (41,51)	19 (37,25)	
	3º trimestre	9 (16,98)	6 (11,76)	
Planejamento da gravidez (n, %)	Sim	21 (39,62)	23 (45,10)	0,572^¶^
	Não	32 (60,38)	28 (54,90)	
Aceitação da gravidez (n, %)	Sim	48 (90,57)	49 (96,08)	0,262^¶^
	Não	5 (9,43)	2 (3,92)	
Nº de consultas (m ± DP)		3,94±2,37	3,51±2,49	0,228^||^

GI - grupo intervenção; GP - grupo placebo; m - média; DP - desvio padrão;

|| Mann-Whitney;

¶ qui-quadrado.

Observou-se que não houve diferença estatisticamente significativa da ansiedade entre os grupos na avaliação final e *follow-up* (Tabela 2). Porém, ao longo do tempo, houve redução estatisticamente significativa da média obtida no IDATE-E entre as avaliações inicial e final no GI (p=0,019) e avaliação inicial e *follow-up* no GP (p=0,001) (Tabela 2).

**Tabela 2:** Análise da ansiedade, verificada pelo Inventário de Ansiedade Traço-Estado, qualidade de vida, verificada pelo *World Health Organization Quality of Life-Bref*, e percepção do medo da COVID-19, verificada pela Escala do Medo da COVID-19, expressas em média, erro padrão e Intervalo de Confiança de 95%, de acordo com o modelo de *Generalized Estimating Equations*, Montes Claros, Minas Gerais, Brasil, 2024 (n=104)

Variável desfecho	Grupo	Inicial	Final	*Follow-up*	Valor de *p*		
		m(ep)	m(ep)	m(ep)	Inicial-final	Final-*follow-up*	Inicial-*follow-up*
IDATE-E	GI	50,36(0,63) ^‡§^	47,92(0,96)^[Table-fn TFN10] ‡§^	48,00(0,94)^[Table-fn TFN10] ‡§^	**0,019^||^**	1,000	0,073
	GP	50,82(0,56)^[Table-fn TFN10] ‡§^	49,04(0,82)^[Table-fn TFN10] ‡§^	48,49(0,88)^[Table-fn TFN10] ‡§^	0,05	0,841	**0,001^||^**
	Valor de *p*	0,581	0,377	0,703			
WHOQOL-percepção qualidade de vida	GI	71,23(2,53)^[Table-fn TFN10] ‡§^	77,83(2,37)^[Table-fn TFN10] ‡§^	75,94(2,40)^[Table-fn TFN10] ‡§^	**0,041^||^**	0,281	0,233
	GP	72,06(1,79)^[Table-fn TFN10] ‡§^	75,49(1,90)^[Table-fn TFN10] ‡§^	74,02(1,83)^[Table-fn TFN10] ‡§^	0,185	0,752	0,938
	Valor de *p*	0,788	0,441	0,524			
WHOQOL-satisfação com a saúde	GI	69,23(3,09)^[Table-fn TFN10] ‡§^	75,00(2,31)^[Table-fn TFN10] ‡§^	73,11(2,48)^[Table-fn TFN10] ‡§^	0,106	0,938	0,726
	GP	69,00(2,96)^[Table-fn TFN10] ‡§^	71,08(2,81)^[Table-fn TFN10] ‡§^	71,77(2,50)^[Table-fn TFN10] ‡§^	1,000	1,000	0,829
	Valor de *p*	0,957	0,281	0,661			
WHOQOL-domínio físico	GI	59,57(2,11)^[Table-fn TFN10] ‡§^	70,82(1,79)^[Table-fn TFN10] ‡§^	70,22(1,86)^[Table-fn TFN10] ‡§^	**<0,001^||^**	1,000	**<0,001^||^**
	GP	62,25(2,29)^[Table-fn TFN10] ‡§^	63,59(2,29)^[Table-fn TFN10] ‡§^	63,94(2,30)^[Table-fn TFN10] ‡§^	1,000	1,000	0,761
	Valor de *p*	0,389	**0,013^||^**	**0,034^||^**			
WHOQOL-domínio psicológico	GI	59,83(2,57)^[Table-fn TFN10] ‡§^	67,45(2,14)^[Table-fn TFN10] ‡§^	66,43(2,04)^[Table-fn TFN10] ‡§^	**<0,001^||^**	1,000	**0,005^||^**
	GP	57,76(2,20)^[Table-fn TFN10] ‡§^	62,50(2,40)^[Table-fn TFN10] ‡§^	61,76(2,41)^[Table-fn TFN10] ‡§^	**0,016^||^**	1,000	**0,019^||^**
	Valor de *p*	0,542	0,123	0,139			
WHOQOL-domínio social	GI	66,03(2,25)^[Table-fn TFN10] ‡§^	69,65(2,36)^[Table-fn TFN10] ‡§^	69,03(2,38)^[Table-fn TFN10] ‡§^	0,302	1,000	0,554
	GP	65,50(2,40)^[Table-fn TFN10] ‡§^	68,17(2,78)^[Table-fn TFN10] ‡§^	69,33(2,65)^[Table-fn TFN10] ‡§^	0,726	1,000	0,162
	Valor de *p*	0,873	0,683	0,931			
WHOQOL-domínio ambiental	GI	61,50(1,83)^[Table-fn TFN10] ‡§^	65,50(1,69)^[Table-fn TFN10] ‡§^	65,80(1,69)^[Table-fn TFN10] ‡§^	**0,010^||^**	1,000	**0,008^||^**
	GP	57,91(1,84)^[Table-fn TFN10] ‡§^	58,95(1,96)^[Table-fn TFN10] ‡§^	58,10(2,13)^[Table-fn TFN10] ‡§^	1,000	1,000	1,000
	Valor de *p*	0,166	**0,011^||^**	**0,005^||^**			
EMC19-9	GI	29,15(0,89)^[Table-fn TFN10] ‡§^	26,47(0,95)^[Table-fn TFN10] ‡§^	26,00(0,92)^[Table-fn TFN10] ‡§^	**<0,001^||^**	0,331	**<0,001^||^**
	GP	28,31(0,82)^[Table-fn TFN10] ‡§^	26,43(0,83)^[Table-fn TFN10] ‡§^	26,41(0,85)^[Table-fn TFN10] ‡§^	**<0,001^||^**	1,000	**<0,001^||^**
	Valor de *p*	0,489	0,975	0,742			

GI - grupo intervenção; GP - grupo placebo;

‡ m - média;

§ ep - erro padrão;

|| p<0,005; WHOQOL-Bref - World Health Organization Quality of Life-Bref; EMC19-9 - Escala do Medo da COVID-19; IDATE-E - Inventário de Ansiedade Traço-Estado.

Em relação à autopercepção da qualidade de vida, não houve diferença estatística significativa da média entre os grupos nos três momentos de avaliação. Mas houve melhora estatisticamente significativa da média do GI entre as avaliações inicial e final (p= 0,041). Na satisfação com a saúde, não houve diferença estatística significativa da média entre o GI e GP na avaliação final e *follow-up*, e não houve diferença da média ao longo do tempo dentro de cada grupo (Tabela 2).

No domínio físico do WHOQOL-Bref, houve diferença estatisticamente significativa entre os grupos na avaliação final (p< 0,001) e *follow-up (*p< 0,001), de forma que o GI apresentou melhor pontuação nesse domínio em relação ao GP (diferença de média: 7,23). Ao longo do tempo, houve melhora estatisticamente significativa entre as avaliações inicial e final (p=0,013) e inicial e *follow-up* (p=0,034) apenas no GI (Tabela 2).

No domínio psicológico do WHOQOL-Bref, não houve diferença estatística significativa entre os grupos na avaliação final e *follow-up* (Tabela 2). Mas houve melhora estatisticamente significativa entre as avaliações inicial e final no GI (p< 0,001) e no GP (p=0,016) e entre a avaliação inicial e *follow-up* no GI (p=0,005) e no GP (p=0,019).

No domínio social do WHOQOL-Bref, não houve diferença estatisticamente significativa da média entre os grupos de tratamento ao longo do tempo e entre os grupos. No domínio ambiental do WHOQOL-Bref, houve diferença estatisticamente significativa da média entre os grupos de tratamento na avaliação final (p=0,011) e *follow-up* (p=0,005), de forma que o GI apresentou melhores pontuações nesse domínio em relação ao GP no momento final (diferença de média:6,55) e no *follow-up* (diferença de média: 7,7). Além disso, houve melhora estatisticamente significativa nesse domínio entre as avaliações inicial e final (p=0,010) e avaliação inicial e *follow-up* (p=0,008) no GI (Tabela 2).

Em relação ao medo da COVID-19, avaliado pela EMC19-9, ao longo do tratamento, não houve diferença estatística significativa entre o GI e GP na avaliação final e *follow-up*. Entretanto, houve redução estatística da média do medo da COVID-19 entre as avaliações inicial e final no GI (p<0,001) e no GP (p<0,001) e entre a avaliação inicial e *follow-up* no GI (p<0,001) e no GP (p<0,001) (Tabela 2).

A maioria das gestantes ficou satisfeita ou extremamente satisfeita com a intervenção realizada, independentemente do grupo. Além disso, a maioria das gestantes no GI considerou que a intervenção foi necessária ou totalmente necessária. Das participantes do GP, houve mulheres que não tiveram certeza da necessidade da intervenção e que a consideraram necessária ou totalmente necessária. A maioria das gestantes do GI considerou o estado geral melhor ou muito melhor. As gestantes do GP relataram estar melhor ou sem nenhuma mudança do estado geral (Tabela 3).

**Tabela 3:** Satisfação das gestantes em relação ao tratamento e à necessidade de realização da intervenção, Montes Claros, Minas Gerais, Brasil, 2024 (n=104)

Variáveis		GI (n=53)	GP (n=51)
Satisfação com a intervenção realizada (n, %)	Não tenho certeza	3 (5,66)	10 (19,61)
	Satisfeito	34 (64,15)	33 (64,71)
	Extremamente satisfeito	16 (30,19)	8 (15,69)
Percepção da necessidade da intervenção (n, %)	Não tenho certeza	1 (1,89)	14 (27,45)
	Necessária	39 (73,58)	26 (50,98)
	Totalmente necessária	13 (24,53)	11 (21,57)
Estado geral após tratamento (n, %)	Muito melhor	15 (28,30)	7 (13,73)
	Melhor	36 (67,92)	33 (64,71)
	Nenhuma mudança	2 (3,77)	11 (21,57)

*GI - grupo intervenção; GP - grupo placebo.*

A maioria das gestantes não relatou reações adversas significativas, evidenciando a segurança da intervenção da auriculoterapia. Observa-se que, do total de gestantes avaliadas, 86,54% (n=90) não apresentaram nenhum sintoma ou reação adversa. As demais mulheres que apresentaram sintomas tiveram cefaleia relacionada à fixação no pavilhão auricular no GI, dor no pavilhão auricular no GI e GP e prurido no pavilhão auricular em ambos os grupos (Tabela 4).

**Tabela 4:** Frequência e intensidade dos sintomas ou reações adversas das pessoas que receberam auriculoterapia, Montes Claros, Minas Gerais, Brasil, 2024 (n=14)

Sintomas ou reações adversas (n=14)	GI		GP	
	Frequência n (%)	Intensidade (m±DP)	Frequência n (%)	Intensidade (m±DP)
Cefaleia relacionada à fixação no pavilhão auricular	2 (3,77)	3,50±3,54	0 (0,00)	0,00±0,00
Dor no pavilhão auricular	5 (9,43)	4,40±1,95	1 (1,96)	2,00±0,00
Prurido no pavilhão auricular	2 (3,77)	1,50±0,71	4 (7,84)	6,00±3,74

*GI - grupo intervenção; GP - grupo placebo; m - média; DP - desvio padrão.*

## DISCUSSÃO

A auriculoterapia demonstrou efeito positivo e similar em relação à ansiedade tanto no GI quanto no GP. Porém, no GI, identificou-se efetividade superior no domínio físico e ambiental da qualidade de vida. Ademais, os grupos apresentaram efeitos similares e positivos no domínio psicológico da qualidade de vida e no medo da COVID-19, sem diferença estatística entre os grupos. No domínio social e na percepção da qualidade de vida, não houve diferença estatística em ambos os grupos. A satisfação com a saúde foi maior com a auriculoterapia entre as avaliações inicial e final, mesmo que não se tenha notado diferença estatística entre os grupos.

O presente estudo não demonstrou diferença estatisticamente significativa entre os grupos para ansiedade. Já em ensaio clínico brasileiro, no qual se avaliou o efeito da auriculoterapia comparado a um grupo controle com consultas habituais de enfermagem em 50 gestantes de pré-natal de risco habitual, houve diferença estatística significativa^(24)^. O efeito superior da auriculoterapia sobre o GP e grupo controle também foi observado em ensaio clínico com 102 parturientes com ansiedade^(28)^ e estudo com 150 mulheres submetidas à aspiração uterina no primeiro trimestre^(29)^. Ressalta-se que a população e o cenário do presente estudo possuem diferenças no período gestacional que influenciam os resultados.

Dessa forma, observou-se que a auriculoterapia tem efeito superior aos grupos controles com cuidados habituais, apesar de não ter diferença estatística com os GPs em situações de abortamento^(30,39)^ e de pré-natal de risco habitual, conforme encontrado no presente estudo. Todavia, enfatiza-se que a escuta e a abordagem dos aspectos emocionais realizada pelo enfermeiro demonstraram efeito terapêutico no GP. A redução da ansiedade no GP também pode ser justificada pela aceitabilidade e satisfação com a técnica^(40)^ e pelos mecanismos psicológicos que resultam no alívio da ansiedade, por meio do condicionamento e expectativa pelo tratamento no GP^(41)^.

A auriculoterapia demonstrou efeito significativo no domínio físico da qualidade de vida, assim como resultados de ensaio clínico realizado na Espanha, que avaliou o efeito da auriculoterapia associada aos cuidados obstétricos padrão na atenção primária, constatando aumento de 40,5% neste domínio no grupo auriculoterapia, comparado ao aumento de 8,1% no grupo controle^(42)^.

Em relação ao domínio psicológico da qualidade de vida, o aumento das médias no GI e GP demonstrou que a abordagem da ansiedade, da qualidade de vida e dos medos que permeiam a gestação influencia positivamente esse domínio^(40)^. Na literatura, entre as abordagens com práticas integrativas e complementares, tem-se avaliado a espiritualidade como componente importante do bem-estar psicológico^(23,43)^. No entanto, não foram encontrados outros estudos que avaliassem o efeito da auriculoterapia especificamente nesse domínio.

Acredita-se que o domínio social não sofreu variações por estar associado a fatores externos à terapia, que inclui as relações sociais, cuidado, amor, conforto, sexualidade, boa relação conjugal, familiar e com amigos^(44)^. Nesse contexto, destaca-se que o apoio social à gestante tem correlação positiva com a qualidade de vida, e é considerado um fator de proteção para ansiedade^(45)^ e medo da COVID-19^(12)^.

O domínio ambiental que, entre as questões, avalia recursos financeiros, disponibilidade e qualidade dos cuidados sociais e de saúde, apresentou diferença estatística significativa para o grupo auriculoterapia. Na literatura, observou-se que a questão econômica é um dos principais fatores negativos da qualidade de vida^(46,47)^ e da ansiedade^(7,48)^. Mas é possível notar que o cuidado em saúde é capaz de melhorar os escores desse domínio, tendo em vista que as gestantes do grupo auriculoterapia demonstraram estar mais satisfeitas e com maiores índices de percepção de que a intervenção foi necessária ou totalmente necessária.

Na literatura nacional e internacional, há carência de evidências quanto ao impacto das PICS no medo da COVID-19. Todavia, na população geral da Turquia, 39,3% fizeram uso dessas práticas na pandemia, sendo a acupuntura utilizada por 3,7% dessas pessoas^(20)^. No presente estudo, no GI e GP, o medo da COVID-19 reduziu de muito para moderado, reforçando que a escuta e a abordagem do medo impactam positivamente a condição^(40)^.

Em relação aos eventos adversos da auriculoterapia, observaram-se cefaleia, dor e prurido no pavilhão auricular com intensidade leve a moderada. Não foram relatados desfechos maternos e fetais com o uso da auriculoterapia, assim como ensaio clínico que avaliou o efeito da auriculoterapia na dor lombar em gestantes^(42)^. Dessa forma, essa e outras evidências têm demonstrado que, assim como a acupuntura sistêmica^(49)^, a auriculoterapia é segura tanto para a mãe quanto para o feto^(24,42)^.

### Limitações do estudo

Entre as limitações do estudo, estão as particularidades socioeconômicas e culturais da população estudada, observadas na homogeneidade da caracterização clínica, obstétrica e sociodemográfica das gestantes, o que torna os resultados não generalizáveis para outras realidades. Além disso, apesar de o IDATE-E ser amplamente utilizado em estudos com gestantes, a ausência de um instrumento traduzido e validado de avaliação da ansiedade relacionada à gestação pode ter influenciado os resultados dessa variável na pesquisa.

### Contribuições para as áreas da enfermagem, saúde ou políticas públicas

O presente estudo contribuiu para a ciência e a área da enfermagem, pois foi demonstrada a segurança da auriculoterapia para ansiedade na gestação, utilizando-se um dispositivo não invasivo. Além disso, a maioria das gestantes, independentemente do grupo de alocação, considerou-se satisfeita ou muito satisfeita e que o tratamento foi necessário ou totalmente necessário, provavelmente devido à deficiência de uma abordagem da saúde mental durante o pré-natal e o foco nos aspectos físicos da gestação. Tais achados reforçam a necessidade do enfermeiro e da equipe multiprofissional rastrearem condições, como ansiedade, qualidade de vida e medo da COVID-19, e implementarem medidas de prevenção e controle, como auriculoterapia.

Portanto, acredita-se que este estudo poderá contribuir para o cuidado de gestantes de baixo risco acompanhadas na APS, além de ser fundamental para futuros estudos em diferentes contextos e tamanhos amostrais. Além disso, colabora no julgamento clínico acerca da ansiedade nessa população e, consequentemente, no impacto de uma assistência de enfermagem de maior qualidade baseada em fortes evidências científicas. Assim, recomendam-se a aquisição de insumos e a capacitação de profissionais inseridos no contexto da APS para utilização da técnica como instrumento capaz de colaborar na redução da ansiedade e melhora da qualidade de vida das gestantes.

## CONCLUSÕES

A auriculoterapia demonstrou efeito similar ao GP para o tratamento da ansiedade em gestantes de risco habitual. Todavia, demonstrou efeito superior ao GP nos domínios físico e ambiental da qualidade de vida. O impacto do efeito nesses domínios da qualidade de vida poderá contribuir para o avanço do conhecimento da área da saúde e enfermagem sobre efeitos da auriculoterapia nessa população e contexto. Além disso, a técnica se demonstrou segura, sem descrição de eventos adversos graves pelas gestantes que participaram do estudo. Por fim, os resultados apontam que o protocolo de auriculoterapia adotado pode ser benéfico na prática clínica, e recomenda-se a realização de investigações futuras em diferentes contextos.
